# Epigenetic ageing during the COVID-19 pandemic: global age acceleration, independent of SARS-CoV-2 infection

**DOI:** 10.1186/s13148-026-02089-x

**Published:** 2026-05-26

**Authors:** Christopher J. Shore, Marc F. Österdahl, Nicholas R. Harvey, Naisi Zhao, Mark Kristiansen, Dominique S. Michaud, Claire J. Steves, Jordana T. Bell, Emma L. Duncan

**Affiliations:** 1 Department of Twin Research and Genetic Epidemiology, King’s College London, London, UK; 2 Department of Ageing & Health, Guy’s and St Thomas’ NHS Foundation Trust, London, UK; 3 Department of Health Sciences and Medicine, Bond University, Robina, QLD Australia; 4 Department of Biostatistics & Data Science, University of Kansas Medical Center, Kansas City, USA; 5 Department of Public Health & Community Medicine, Tufts University School of Medicine, Medford, USA; 6 UCL Genomics, University College London, London, UK; 7 Genetics and Genomic Medicine Department, UCL Great Ormond Street Institute of Child Health, London, UK; 8 Department of Endocrinology, Guy’s and St Thomas’ NHS Foundation Trust, London, UK

**Keywords:** COVID-19, Lockdown, Epigenetic ageing, Epigenetic clocks

## Abstract

**Introduction:**

The COVID-19 pandemic had profound effects for both infected and uninfected individuals. We aimed to identify acute and chronic epigenetic alterations from SARS-CoV-2 infection and/or lockdown measures.

**Methods:**

Both infected and uninfected participants from the TwinsUK cohort (*n* = 139) provided longitudinal whole-blood samples (Spring 2020, Spring 2021). Cross-sectional and longitudinal DNA methylome [DNAm] changes, including epigenetic ageing clocks, were assessed, and EWAS (Epigenome-Wide Association Studies) were performed. Changes in epigenetic ageing clocks were compared with longitudinal samples from an independent pre-pandemic cohort (*n* = 35, Nurses’ Health Study II cohort), assessed similarly.

**Results:**

Irrespective of SARS-CoV-2 infection, all individuals exhibited accelerated ageing from 2020 to 2021 (GrimAge clock age acceleration: p-value = 0.002, β = 0.99). In contrast, epigenetic data from paired samples from a separate pre-pandemic cohort, over a similar time period and assessed similarly, did not exhibit GrimAge acceleration. Epigenetic ageing did not differ between infected and uninfected individuals, or between recent and distant SARS-CoV-2 infection. Lastly, EWAS identified several putative changes in DNAm associated with recent, and distant, SARS-CoV-2 infection.

**Conclusions:**

Our longitudinal results suggest that the pandemic accelerated epigenetic ageing rates, irrespective of recent or distant SARS-CoV-2 infection.

**Supplementary Information:**

The online version contains supplementary material available at 10.1186/s13148-026-02089-x.

## Introduction

The COVID-19 pandemic caused profound population effects, both from SARS-CoV-2 infection and from lockdown. Social upheaval in locked-down populations included decreased physical activity, increased sedentary behaviour [33], altered dietary habits [2], increased loneliness [6], and deteriorated mental health [23].

The epigenome is closely associated with ageing, morbidity, and mortality, both in animal models and humans [19]. Epigenetic ‘clocks’ exploit this association, using DNA methylation [DNAm] patterns to estimate ageing. First generation clocks (e.g., Hannum [11], Horvath [13], showed close association of DNAm with chronological age. Second generation clocks (e.g., GrimAge [20], PhenoAge [18] incorporated epigenetic changes associated with morbidity and mortality, capturing ‘biological’ age. These in turn could be used to illuminate discrepancies between predicted ‘biological’ age and chronological age, to estimate biological age ‘acceleration’.

Several studies have examined DNAm patterns following SARS-CoV-2 infection (recently reviewed [8]. However, most studies were cross-sectional and focused on DNAm changes following acute infection. Further, most studies assessed mainly hospitalised individuals with severe COVID-19. However, most cases of SARS-CoV-2 infection, even pre-vaccination, were mild, and managed in the community. Additionally, many pandemic-related environmental changes were known pre-pandemic to be associated with accelerated epigenetic ageing [9]. Few studies have examined longitudinal epigenetic changes in uninfected individuals across the pandemic.

We hypothesized that the pandemic may have accelerated epigenetic ageing, whether through SARS-CoV-2 infection, lockdown effects, or both. Thus, we aimed to assess firstly whether there was a pandemic-related effect on epigenetic ageing rates; and secondly whether SARS-CoV-2 infection influenced epigenetic ageing, for either recent and/or distant infection. Lastly, we tested for SARS-CoV-2 infection effects on DNAm patterns across the genome.

### Methods

### Discovery cohort

This study was carried out under TwinsUK BioBank ethics, approved by North West – Liverpool Central Research Ethics Committee (REC reference 19/NW/0187), IRAS ID 258,513. Study participants were drawn from the TwinsUK research cohort [32]. Individuals in the current study comprised of participants who were based in London or in close proximity, because of pandemic travel restrictions.

Blood samples were collected in Spring 2020 (April-June 2020, with wild-type the predominating SARS-CoV-2 variant) and approximately a year later in Spring 2021 (April/May, with alpha the predominating variant) (UK Health Security Agency (UKHSA), [29]). Self-reported SARS-CoV-2 infection and vaccination status in these individuals were assessed as previously described [4] (Supplementary Methods).

Smoking status was determined using cg05575921 methylation status (a strong predictor of current smoking status [3], as pre-pandemic data had suggested unreliability of self-reported smoking. BMI was derived from either pre-pandemic TwinsUK clinical visit assessments (95.3%) or self-reported height and weight (4.7%) (median time gap for Spring 2020 sample collection: 3 years, range 0–7 years).

**Fig. 1:**
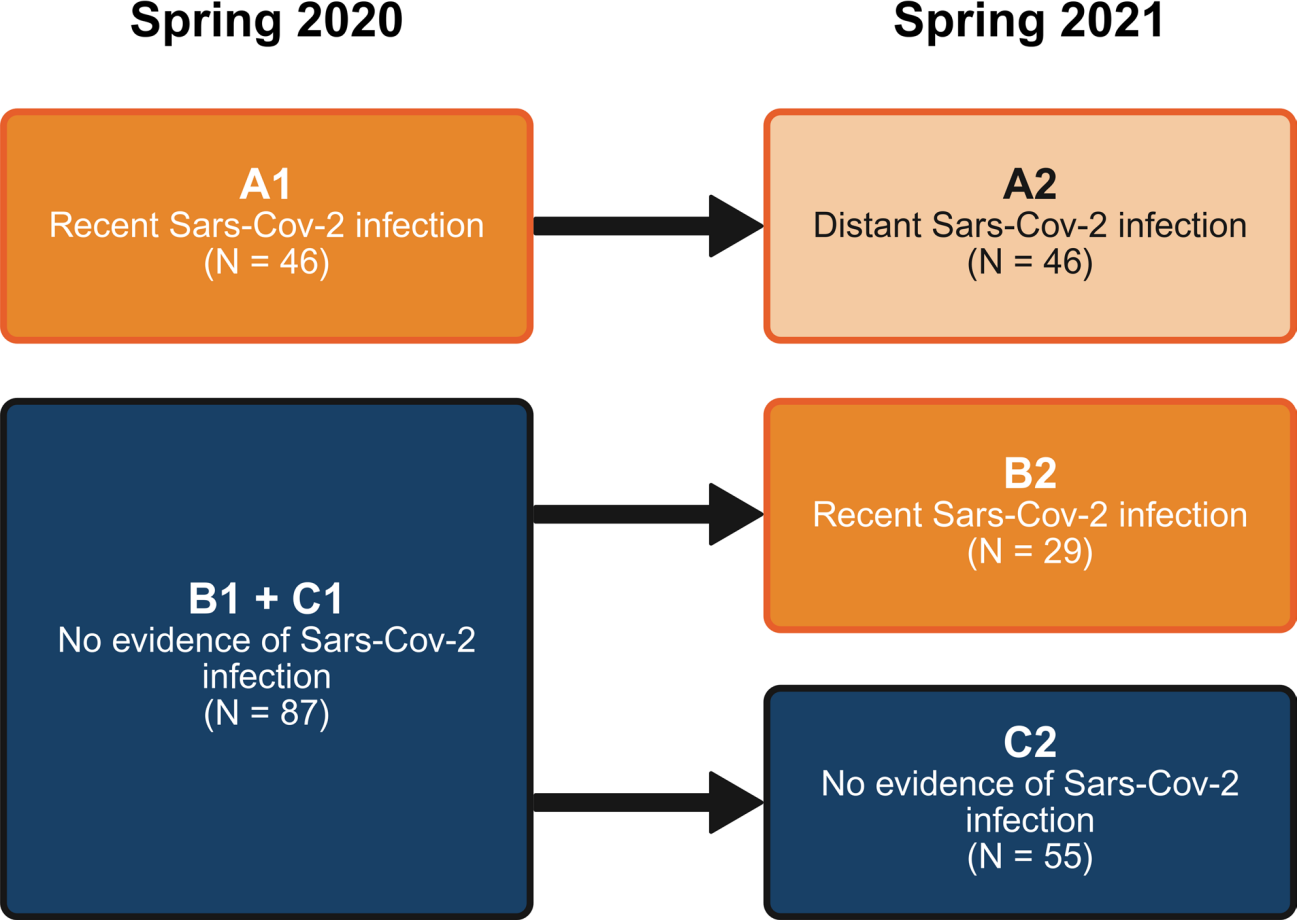
TwinsUK cohort infection status classification

Participants were categorised as per Fig. 1. The first group comprised individuals infected in Spring 2020 (Fig. 1: A1/A2) We note that these individuals could experience subsequent re-infection, however this information was not captured. The second group included individuals not infected in Spring 2020 (Fig. 1: B1), who became infected by Spring 2021 (Fig. 1: B2). Note that these individuals could have been infected at any time during the preceding year. The third group comprised individuals who were neither infected in Spring 2020 (Fig. 1: C1) nor by Spring 2021 (Fig. 1: C2).

### DNA methylation profiling

Bisulphite-converted DNA samples extracted from whole-blood were epigenetically profiled using the Illumina HumanMethylation EPIC v1 BeadChip platform (Illumina, San Diego, CA). To reduce confounding of batch effects with sample year, DNA from samples collected in 2020 and 2021 were assessed together; and were randomised for array placement (such that each Illumina array could include samples from both 2020 and 2021). DNAm profiles underwent quality control assessments and normalisation using ENmix R software [34]. Briefly, after excluding probes for missingness (probe detection p-value > 0.05 in > 5% of samples) and/or high likelihood of off-target mapping [36], DNAm profiles were normalised for background correction and probe-type bias (see Supplementary Methods).

The DNA Methylation Age Calculator website [13] was used to generate epigenetic age- and age-acceleration estimates, using GrimAge [20] and Horvath [13] epigenetic clocks.

### Estimation of blood cell-type composition

DNAm-based cell-type composition estimates for neutrophils, monocytes, B cells, Natural Killer (NK) cells, CD8 + T cells, and CD4 + T cells were generated using the *estimateCellCounts* function of the minfi R package [1], per previously published methods [14].

### Effect of pandemic on epigenetic ageing

To assess longitudinal epigenetic aging, DNAm patterns were compared between paired blood samples collected in Spring 2020 and Spring 2021. Specifically, GrimAge age acceleration values were fitted using a linear mixed model with fixed effects of sample year, cg05575921-assessed smoking status, all cell-type composition estimates, and chip row; and random effects of relatedness, participant ID, and chip. This model was compared to a null model, omitting year of sample collection, using ANOVA. Sensitivity analyses were performed with additional covariates BMI and SARS-Cov-2 infection status.

### Validation cohort and longitudinal epigenetic ageing comparison

To compare epigenetic ageing rates during the pandemic with epigenetic ageing rates before the pandemic, we explored previously published longitudinal DNAm profiles obtained approximately 7 years prior to the pandemic in an independent healthy female sample of similar age (Nurses’ Health Study II samples) [35]. Briefly, DNAm was explored using the Illumina MethylationEPIC [850 K] BeadArray in paired whole blood samples from 35 healthy women enrolled in the Nurses’ Health Study II cohort (baseline mean age (SD) = 61 years (3.5), mean BMI (SD) = 27 (5.2), 69% never smokers). The samples were collected approximately 1 year apart from 2013 to 2014. We repeated the epigenetic ageing analyses above (omitting adjustment for relatedness) using these pre-pandemic blood DNAm.

### Effect of SARS-CoV-2 infection on epigenetic age

To address whether *recent* infection affected epigenetic ageing profiles, we compared DNAm profiles of infected vs. uninfected individuals using three approaches. First, we performed a cross-sectional analysis of 2020 profiles only (Fig. 1: A1 vs. [B1 + C1]). Second, we repeated this cross-sectional analysis, but using 2021 profiles only (Fig. 1: B2 vs. C2). Third, we performed a cross-sectional analysis of the full dataset using both 2020 and 2021 profiles, adjusting for the inclusion of two profiles for most individuals as described below, and excluding 2021 profiles from individuals infected by Spring 2020 (Fig. 1: [A1 + B2] vs. [B1 + C1 + C2]).

To address whether *distant* infection affected epigenetic ageing profiles, we used two approaches. First, we compared the effect of distant infection with recent infection, by performing both a longitudinal analysis of individuals infected before Spring 2020 (Fig. 1: A2 vs. A1). Second, we performed a cross-sectional analysis of 2021 profiles which compared individuals infected before Spring 2020 with individual only infected between Spring 2020 and 2021 (Fig. 1: A2 vs. B2).

For each of the above, GrimAge acceleration values were fitted to a linear-mixed model adjusting for age, sex, cg05575921-assessed smoking status, cell counts, and chip row as fixed effects, and relatedness, chip, and participant ID (only in analyses including multiple samples from the same individual) as random effects. We also performed sensitivity analyses by additionally including BMI as a fixed effect covariate. For analyses using data from 2021 only, we also performed an additional sensitivity analysis adjusting for vaccination status as a fixed effect. Analyses were also repeated using the Horvath Epigenetic Ageing Clock [13], which assesses predominantly chronological age.

### Epigenome-wide association studies

We considered DNAm variation at individual genomic sites in relation to SARS-CoV-2 infection. Epigenome-wide association studies (EWAS) were conducted with three approaches.

First, we assessed the effect of recent infection vs. no infection on DNA methylation. To this end we performed an EWAS of infection status based on Spring 2020 profiles only (Fig. 1: A1 vs. [B1 + C1]), and an EWAS of infection status in Spring 2021 profiles only, excluding individuals infected before Spring 2020 (Fig. 1: B2 vs. C2). We also performed an EWAS of recent infection vs. no infection, including both Spring 2020 and Spring 2021 profiles, accounting for repeated measurements of the same individuals as described below (Fig. 1: [A1 + B2] vs. [B1 + C1 + C2]). This third EWAS was performed to maximise statistical power and minimise confounding from effects occurring across the pandemic (which might occur if comparing B1 to B2, without also including C1 and C2).

Second, we assessed the effect of distant vs. recent infection on DNAm by performing two EWAS. The first of these was a longitudinal EWAS comparing 2020 and 2021 profiles from individuals who were infected prior to Spring 2020 (Fig. 1: A1 vs. A2). The second was a cross-sectional EWAS, comparing 2021 profiles of distantly infected individuals with recently infected individuals (Fig. 1: A2 vs. B2).

Third, we assessed the effect of distant vs. no infection on DNAm, using a cross-sectional EWAS of Spring 2021 profiles only (Fig. 1: A2 vs. C2).

Each EWAS were performed by fitting rank-normalised beta values for each CpG site to a linear mixed model, which included either SARS-CoV-2 infection status as a predictor or - depending on the specific EWAS - a variable specifying ‘recent’ or ‘distant’ infection. This ‘fit’ model included cell-type composition, age, sex, smoking status, and DNAm profiling chip row as fixed effects; and relatedness and DNAm profiling chip as random effects. In analyses including multiple profiles from the same individual, subject ID was also included as a random effect. The ‘fit’ model for each CpG site was then compared using ANOVA to a ‘null’ linear mixed model, which omitted the predictor (i.e. SARS-CoV-2 infection status or recent/distant infection) but did include all fixed and random covariates, to obtain a p-value for the predictor’s effect on the methylation status of the CpG site.

Statistical analyses were performed using R (version 4.3). EWAS test statistics and p-values were adjusted for inflation and bias using the bacon R package [30]. Subsequently, bacon-adjusted p-values were corrected for multiple testing using the Bonferroni false discovery rate. Per previous EWAS with similar sample sizes [12, 17], significant association was defined as bacon- and Bonferroni-adjusted p-value < 0.05.

## Results

### Cohort description

The cohort comprised 137 TwinsUK research participants with blood-derived DNA samples and serology data collected in both Spring 2020 and Spring 2021. After quality control, 129 individuals had DNAm profiles for both timepoints and eight individuals had DNAm profiles for one timepoint only. The mean time difference between longitudinal sample collection was 336 days or approximately 11 months.

Discovery sample demographic characteristics are shown in Table 1. Reflecting the TwinsUK cohort, the population was predominantly female (86.9%) and middle-aged (2020 mean age: 50.7 years). Few individuals (6 [4%]) were current smokers in Spring 2020, based on DNAm profiles. By Spring 2021, 75 of 133 individuals (56%) had received at least one vaccination against SARS-CoV-2. Three individuals had uncertain serological evidence of previous SARS-CoV-2 infection in Spring 2021 and were excluded from analyses examining SARS-CoV-2 infection. BMI data was recorded prior to the COVID-19 pandemic and was missing for 10 individuals.

**Table 1:** Baseline demographics of discovery cohort. Numbers vary slightly due to differential sample missingness at different timepoints

	Total Cohort (Spring 2020)	2020		2021		
		SARS-CoV-2 positive in Spring 2020 (i.e., recent infection in 2020) (A1)	SARS-CoV-2 -negative in Spring 2020 (i.e., no infection in 2020) (B1 + C1)	SARS-CoV-2 positive in Spring 2020 (i.e., distant infection) (A2)	SARS-CoV-2 negative in 2020 but positive in 2021 (i.e., recent infection in 2021) (B2)	SARS-CoV-2 negative in Spring 2020 and negative in Spring 2021 (i.e., never infected) (C2)
N	137	46	87	46	29	55
Mean Age (SD)	50.7 (16.0)	49.9 (15.7)	50.9 (16.1)	50.2 (16.1)	49.4 (15.0)	52.7 (16.9)
Female (%)	119 (86.9%)	38 (82.6%)	79 (90.8%)	38 (82.6%)	27 (93%)	49 (89.1%)
Current Smokers (cg05575921-assessed) (%)	6 (4.4%)	1 (2.2%)	5 (5.8%)	1 (2.2%)	1 (3.4%)	3 (5.5%)
Mean BMI (SD)	25.2 (5.1)	25.7 (5.8)	25.1 (4.8)	25.7 (5.8)	23.7 (4.1)	25.6 (5.1)
N Vaccinated	–	–	–	24 (52.2%)	17 (58.6%)	34 (61.8%)

### Accelerated epigenetic ageing from 2020 to 2021

**Fig. 2:**
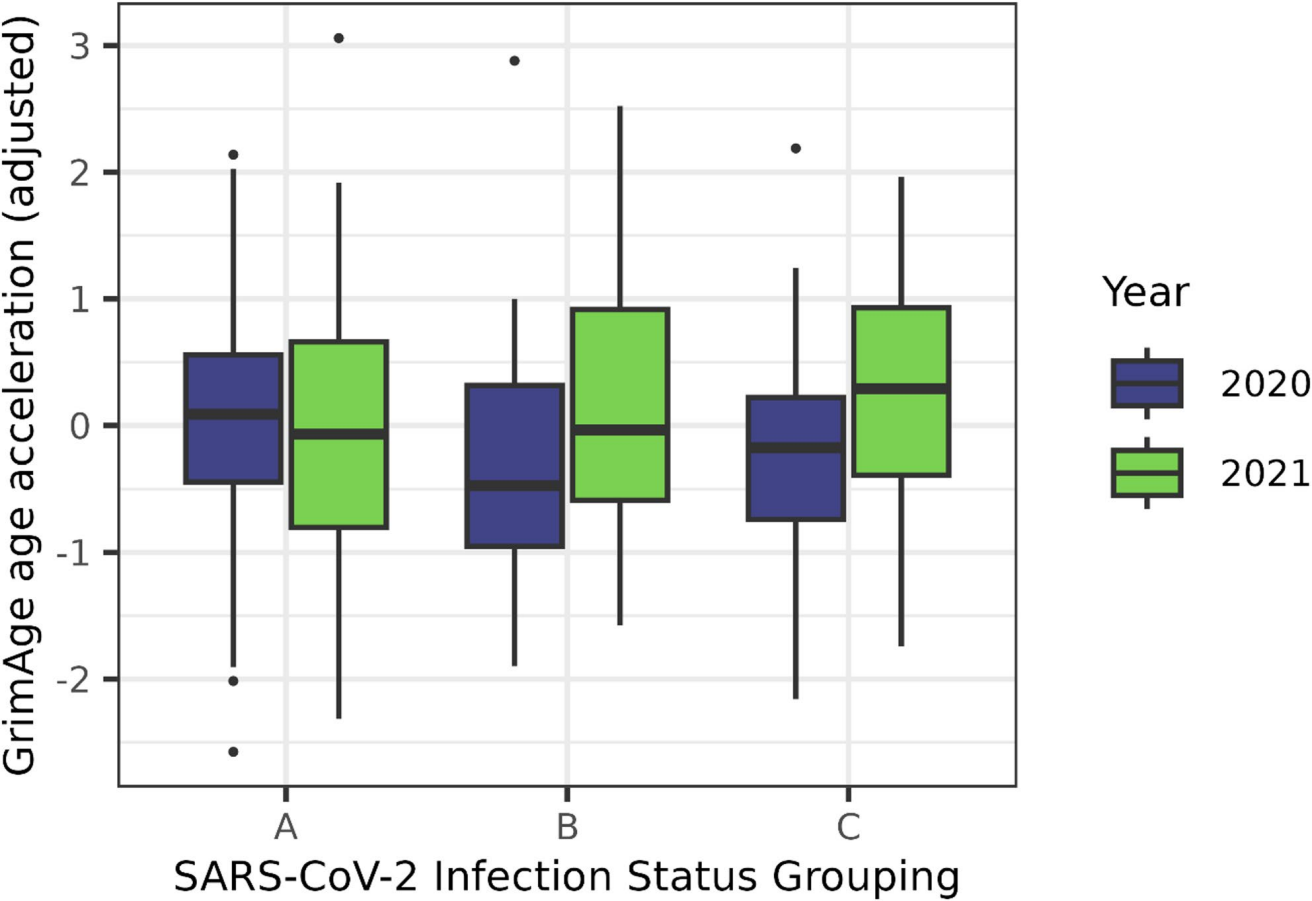
Changes in GrimAge acceleration from Spring 2020 to Spring 2021. GrimAge acceleration values have been adjusted for covariates in a linear mixed model (see Methods). Samples are categorised within ‘A’, ‘B’, and ‘C’ groups according by SARS-CoV-2 infection status, as described in Fig. 1. Specifically, ‘A’ represents individuals infected prior to Spring 2020 sample collection, ‘B’ represents individuals first infected between Spring 2020 and Spring 2021 sample collection, and ‘C’ represents individuals with no evidence for infection by Spring 2021

Considering epigenetic ageing rate from Spring 2020 and Spring 2021 in 129 individuals with data available at both timepoints, and adjusting for relatedness, we observed a significant increase in GrimAge epigenetic age acceleration (beta = 1.6, se = 0.22, *p* = 3.7e-12) (Table S1). After adjustment for additional biological and technical covariates, including cell-type proportions, we still observed an increase in GrimAge epigenetic age acceleration between Spring 2020 and Spring 2021 (beta = 0.99, se = 0.25, *p* = 0.001) (Fig. 2). This result was robust to adjustment for SARS-CoV-2 infection status and BMI (*N* = 119, beta = 0.98, se = 0.25, *p* = 0.002). Interestingly, this effect was not observed in the subset of individuals infected prior to Spring 2020 (participant group A, *N* = 46) when analysed separately (Fig. 2).

We also observed changes in blood cell-type composition estimates between Spring 2020 and Spring 2021. Specifically, there was a nominally significant increase in the proportion of neutrophils (beta = 0.025, *p* = 0.0001), and a decrease in the proportion of monocytes (beta = -0.007, *p* = 2.1e-6), CD8 + T cells (beta = -0.007, *p* = 0.025), and NK cells (beta = -0.009, *p* = 1.2e-5) (Table S3). Interestingly, when examining individuals from only a single infection status grouping (Fig. 1: A, B, or C), the direction of effect in change for these three cell types was consistent, although effects were not always nominally significant (Table S3). We adjusted our model for all cell-type proportions when assessing the longitudinal change in GrimAge acceleration; but to ensure that these four cell-types were not themselves driving the observed effect we performed four sensitivity analyses, adjusting for relatedness, technical covariates, and one of the four cell-type proportions (in turn). Our observation of increased GrimAge acceleration across the pandemic was consistent in all four sensitivity analyses (Table S1\4).

### Longitudinal epigenetic ageing rate during vs. before the pandemic

In contrast to our longitudinal pandemic results, 1-year longitudinal analysis of GrimAge acceleration rates in the Nurses’ Health Study II [35] showed a small deceleration in epigenetic ageing (beta = -0.06, se = 0.01, *p* = 0.0002). Additionally, unlike in the TwinsUK cohort, we did not observe any significant changes in blood cell-type composition estimates over the 1-year period (Table S5).

### Effect of recent SARS-CoV-2 infection on epigenetic ageing rate

After adjusting for biological and technical covariates, there was no detectable effect of recent SARS-CoV-2 infection on GrimAge acceleration (Table 2). This includes after additional adjustments for BMI and vaccination status (Table S2).

**Table 2:** The effect of recent SARS-CoV-2 infection on GrimAge age acceleration. Results in Row 3 including profiles from individuals in groups B and C, with the aim of maximising statistical power and minimising any confounding from the observed changes in age acceleration across the pandemic (which might occur if comparing B1 to B2, without also including C1 and C2) (Fig. 2)

Comparison	β (standard error)	*p*-value
Infection prior to Spring 2020 vs. no infection in Spring 2020 (Fig. 1: A1 vs. [B1 + C1])	0.46 (0.36)	0.21
Infection between Spring 2020 and Spring 2021, vs. no infection in Spring 2021 (Fig. 1: B2 vs. C2)	− 0.12 (0.55)	0.77
Recent infection vs. no infection, with data from both Spring 2020 and Spring 2021 (Fig. 1: [A1 + B2] vs. [B1 + C1 + C2])	0.25 (0.29)	0.41

### Effect of distant SARS-CoV-2 Infection on epigenetic ageing rate

After adjusting for biological and technical covariates, there was no effect of distant SARS-Cov-2 infection on epigenetic age compared to those with recent SARS-Cov-2 infection, or who never experienced SARS-Cov-2 infection (Table 3).

**Table 3:** Effect of time since SARS-CoV-2 infection on GrimAge age acceleration

Comparison	β (standard error)	*p*-value
Recent vs. distant infection (paired data from same individuals) (A1 vs. A2)	− 0.39 (0.43)	0.33
Recent vs. distant infection (cross-sectional analysis using different individuals, at the same time point (A2 vs. B2)	− 0.36 (0.63)	0.54
Distant vs. never infection (cross-sectional analysis using different individuals, same time point (A2 vs. C2)	− 0.15 (0.46)	0.75

### Epigenome-Wide Association Studies (EWAS) of recent and distant SARS-CoV-2 infection

After correction for inflation and multiple testing (see *Methods*), genome-wide significant (bacon- and Bonferroni adjusted p-value < 0.05) epigenetic effects of SARS-CoV-2 infection on whole-blood methylome were seen for:

i) Recent vs. no infection, for Spring 2021 cross-sectional samples only (Fig. 1: B2 vs. C2; 36 signals; Table S6).
ii) Distant vs. recent infection, for both cross-sectional (Fig. 1: A2 vs. B2; 24 signals) and longitudinal (Fig. 1: A1 vs. A2; 11 signals) analyses (Table 4, S7, S8).

Considering Spring 2021 recent vs. no infection analysis, we observed 36 genome-wide significant effects. Of these, seven were in intergenic regions, eleven were in or near gene promoters, fifteen were in gene bodies, and five were in 5’ or 3’ untranslated regions (Table S6). The most strongly associated signal was located in an intron of overlapping genes *UGT2A1* and *UGT2A2* (cg19874327, beta = -0.53, se = 0.05, adjusted *p* = 1.2 × 10^− 17^). Genetic variants in these genes have previously been associated with SARS-CoV-2 infection in GWAS [26]. Several other signals were also located in genes previously related to SARS-CoV-2 infection, including cg04964838 in the gene body of *EIF4A1* (in proteomics analyses [5], and cg13891330 in the promoter of *PPP1R14A* (in immunological analyses [16] (Table S6). No significant results were seen for recent infection in Spring 2020 (Fig. 1: A1 vs. [B1 + C1]) or for the combined Spring 2020 and 2021 profiles (Fig. 1: [A1 + B2] vs. [B1 + C1 + C2]).

Considering distant vs. recent infection we observed significant changes both in the same individuals longitudinally (Fig. 1: A1 vs. A2; 11 signals), and in different individuals cross-sectionally (Fig. 1: B2 vs. A2; 24 signals) (Table 4). The most strongly associated signals were cg21210537 in the promoter region of *MIR769*, and cg05194233, in the first exon of *FBN2*, respectively. Among these 35 unique signals, notable sites included those annotated to the gene body of *PIK3CD* (which has previously been suggested as a therapeutic target for SARS-CoV-2 infection [21] and in the promoter region of *DEGS1* (which has been linked to SARS-CoV-2 infection severity [28] (Table S7, S8).

Altogether, there were 71 unique signals identified related to recent or distant SARS-CoV-2 infection that annotate to 64 unique genes (Tables S6-S8). None of these CpG sites were significant in more than one EWAS, although cg07374121, in the promoter region of *PIK3CD*, and cg15192374, in the gene body of *PIK3CD*, were significant in the EWAS of recent vs. no infection (Fig. 1: B2 vs. C2), and the longitudinal EWAS of recent vs. distant infection (Fig. 1: A1 vs. A2). The EWAS of distant infection vs. no infection did not identify any significant associations.

**Table 4:** Number of CpG sites significant in EWAS of distant or recent SARS-CoV-2 infection. Results in Row 3 including profiles from individuals in groups B and C, with the aim of maximising statistical power and minimising any confounding from the observed changes in age acceleration across the pandemic (which might occur if comparing B1 to B2, without also including C1 and C2) (Fig. 2)

Comparison	Number of significantly associated CpG sites
Recent vs. no infection (cross-sectional analysis at Spring 2020) (Fig. 1: A1 vs. (B1 + C1))	0
Recent vs. no infection (cross-sectional analysis at Spring 2021) (Fig. 1: B2 vs. C2)	36
Recent vs. no infection: combined Spring 2020 and 2021 profiles (Fig. 1: [A1 + B2] vs. [B1 + C1 + C2]).	0
Distant vs. recent infection (longitudinal analysis in the same individuals) (Fig. 1: A1 vs. A2)	11
Distant vs. recent infection (cross-sectional analysis) (Fig. 1: A2 vs. B2)	24
Distant infection vs. never infected (cross-sectional analysis) (Fig. 1: A2 vs. C2)	0

## Discussion

Here we examined the impact of the pandemic on the blood DNA methylome in longitudinal samples from the TwinsUK cohort. Between Spring 2020 and Spring 2021, individuals demonstrated accelerated epigenetic ageing, measured using the GrimAge clock. However, SARS-CoV-2 infection *per se* did not have an independent effect in our cohort. This suggests that accelerated epigenetic ageing was likely due to pandemic-related physical, social, psychological and/or environmental stress. Our EWAS also identified multiple CpG sites associated with either recent or distant SARS-CoV-2 infection status.

Previous studies examining epigenetic ageing and the pandemic have been mainly cross-sectional, and usually in individuals with severe COVID-19, with varying results. Most cross-sectional studies report increased epigenetic age acceleration (recently reviewed by [15], but these were primarily assessing severe illness requiring hospitalisation - in contrast our cohort were community-managed. A recent longitudinal study in 54 individuals (of whom only half self-reported experiencing COVID-19 by the end of 2022) identified both accelerated and decelerated epigenetic ageing, depending on which of eight epigenetic clocks was used. Notably, this study identified an increase in GrimAge acceleration over the pandemic only if assessing individuals who were infected with SARS-CoV-2 between time points [7]. Stressor events, including severe COVID-19, can cause transient epigenome changes that can revert on recovery [24]; thus, timing of infection relative to sample collection may contribute to these varying results. SARS-Cov2-infection also affects blood cell composition [22], which may also contribute to heterogenous results in different studies particularly if these use different clocks (noting here that cell-type composition has been usually unreported in previous studies).

The pandemic changed behaviour across populations, with greater sedentariness, less adherence to healthy eating guidance, and sleep disturbance [10, 25, 27] was a time of huge emotional and psychological stress across the population. Pre-pandemic studies showed all these behavioural changes are associated with epigenetic changes [31], which may be of relevance for our own findings.

Our EWAS identified epigenetic changes associated with recent SARS-CoV-2 infection status, and with distant vs. recent SARS-CoV-2 infection. However, we only identified significant changes for recent infection when assessing samples from Spring 2021, and neither for samples from Spring 2020 nor when analysing both time points together. This could suggest that epigenetic changes induced by recent SARS-CoV-2 infection are associated with specific variants of SARS-CoV-2, and/or that they are simply false positive results. However, our EWAS was able to identify loci previously associated with SARS-CoV-2 infection. Perhaps most notably, epigenetic changes in *PIK3CD* were associated with recent SARS-CoV-2 infection, and with recent vs. distant SARS-CoV-2 infection - although the specific CpG site differed between these two analyses. The protein product of *PIK3CD* is in the PI3Kδ family, heavily involved in immune regulation in leukocytes, and previously identified as potential therapeutic targets against SARS-CoV-2 infection [21]. These epigenetic changes could be evidence of altered transcriptional activity of *PIK3CD* in SARS-CoV-2 infection; further analysis of these effects could validate the therapeutic potential of PI3Kδ pathways. Lastly, our findings of epigenetic changes associated with recent vs. distant SARS-CoV-2 infection suggest possible discrete long-term vs. short-term epigenetic effects of infection; this requires confirmation in further studies and cohorts.

Study strengths include its longitudinal design—rare among pandemic epigenetic studies—and a community-based cohort, reflecting typical SARS-CoV-2 infection experiences. Infection status was confirmed robustly via serology. We applied a validated epigenetic clock to assess biological vs. chronological ageing. Longitudinal sampling during the most restrictive 15 months of the pandemic captured the period of greatest environmental disruption from lockdown and other measures. Our epigenetic method offers a direct and integrative health measure, avoiding the limitations of retrospective self-reporting which are particularly prone to error in stochastic variables like diet and exercise. In addition, we used the GrimAge epigenetic clock, which has been shown to capture age-related mortality and disease better than first generation clocks such as the Horvath DNAmAge clock 20 (which we also analysed (Table S1)). Finally, we compared pandemic and pre-pandemic longitudinal data, over similar timeframes and methods (albeit in different cohorts), reinforcing that our observed changes were pandemic specific.

Limitations include those imposed by pandemic-related travel restrictions, affecting both cohort size and composition (necessarily London-centric, with its specific socioeconomic backgrounds). These geographic constraints also hindered selective recruitment by twin zygosity and/or infection status. Also, as typical for TwinsUK, our cohort was predominantly female. These issues limit generalisability of our findings. While infection status was confirmed serologically, infection timing was unknown, potentially causing variable intervals between infection and sampling. This was less problematic for the first collection, conducted soon after widespread UK infections, but potentially more so for the second collection, when infection could have occurred anytime in the preceding year. Further, we did not assess for repeat infections. These timing issues may obscure distinctions between recent and distant infections. Our small sample size also precluded assessment of individual aspects of the pandemic experience (e.g., diet, behaviour, exercise) which will require a larger cohort. Our small sample size also limits our power to detect potential smaller effects of SARS-CoV-2 infection on GrimAge acceleration, arguably more likely in mild and/or community-managed cases compared with severe and/or hospital-requiring infection. In addition, due to unreliable self-reported smoking status data, we adjusted for smoking status using the methylation status of a single CpG site, cg05575921. This CpG site is one of the 1,030 included in the GrimAge epigenetic clock, and as such could bias our results. However, we observed an increase in GrimAge acceleration from 2020 to 2021 when both including and not including cg05575921 estimated smoking status, suggesting that any potential bias is minimal. We also acknowledge that comparing the Twins UK cohort results to those of the pre-pandemic Nurses’ Health Study II cohort is not ideal, given the latter’s small sample size (*N* = 35) and differing structure and geography. Nonetheless, this timing of this cohort’s longitudinal sample collection (one year apart) matched the study design; the epigenetic profiling used the same technologies; and the age and gender demographics are broadly similar to the Twins UK cohort. We are mindful of other pre-pandemic longitudinal datasets – however, the timing of their longitudinal sample collections differed substantially; and the cohorts were much more demographically distinct.

Lastly, we interpret our EWAS results very cautiously. Our sample sizes are small, limiting power; and we lack an independent replication cohort. Thus although some analyses showed association at previously reported loci, the robustness of our results must be considered, reinforced by the lack of overlap of significant CpG sites between each EWAS. We have also not considered all potential confounding co-morbidities (e.g., other illnesses) although we did adjust for age, sex, smoking and BMI. Although we used an established Bayesian approach to limit false-positive discovery [30], our 2021 recent infection analyses found 36 significant results. This stands in contrast to the number of loci identified in a similarly powered EWAS of SARS-CoV-2 infection status (182 individuals, comparing cases 3 months post-infection (*N* = 109) with uninfected controls (*N* = 73), that identified three CpG sites using a similar Bayesian approach towards adjustment of EWAS test statistics [17]. Further, we found no significant results for recent vs. no infection for Spring 2020 data alone or when we combined the Spring 2020 and Spring 2021 data sets. Whilst we found significant results in both analyses of distant vs. recent infection, there were few shared loci between these datasets, which *a priori* seems biologically unlikely.

In conclusion, we observed accelerated epigenetic ageing during the pandemic in a community cohort of UK twins. SARS-CoV-2 infection *per se* did not have an independent effect; and there was no epigenetic ageing difference when considering distant vs. recent SARS-CoV-2. As the pandemic recedes, it would be interesting to reassess these same individuals, to see whether the accelerated changes observed here regressed over time to concord more closely with chronological ageing.

## Supplementary Information

Below is the link to the electronic supplementary material.

Supplementary Material 1

Supplementary Material 2

## Data Availability

The TwinsUK blood methylome dataset in this manuscript is deposited in the EGA under the accession number EGAS00001008409.
