# Supplementary material for: Epigenetic ageing during the COVID-19 pandemic: global age acceleration, independent of SARS-CoV-2 infection
Source: Clin Epigenetics. 2026 May 26;18:96. doi: 10.1186/s13148-026-02089-x (PMC13202874; doi:10.1186/s13148-026-02089-x)
Supplement: Supplementary file 1 — Supplementary Material 1 [file 13148_2026_2089_MOESM1_ESM.docx]

### Supplementary Methods

### SARS-CoV-2 status assessment relative to vaccination

Participants were considered serologically positive for infection:

1. Before vaccination: if they had an IgG Spike titre fold change ≥4.0 above background,
2. After vaccination: IgG Spike and IgG nucleocapsid titre fold change ≥4.0 above background.

If a participant’s antibody titre was previously positive, but declined on subsequent testing, they were still considered positive for past infection, as previously published (Cheetham et al., 2023).

## DNAm Profiling: QC

Whole-blood DNAm profiles were generated using Illumina HumanMethylation EPIC V.1 BeadChip arrays.

CpG site probes were removed due to missingness at a threshold of probe detection p-value > 0.05 in >5% of samples (N = 27,208).

CpG site probes previously identified to have a high likelihood of off-target mapping (Zhang et al., 2022) were also removed (N = 86,537).

DNAm data was normalised for background correction using the ENmix algorithm (Xu et al., 2016), then quantile normalised across samples, and finally the Regression on Correlated Probes (RCP) algorithm (Niu et al., 2016) was applied to correct for probe/design bias using the ENmix R package (Xu et al., 2016).

References

Cheetham, N. J., Kibble, M., Wong, A., Silverwood, R. J., Knuppel, A., Williams, D. M., Hamilton, O. K., Lee, P. H., Bridger Staatz, C., Di Gessa, G., Zhu, J., Katikireddi, S. V., Ploubidis, G. B., Thompson, E. J., Bowyer, R. C., Zhang, X., Abbasian, G., Garcia, M. P., Hart, D., . . . Steves, C. J. (2023). Antibody levels following vaccination against SARS-CoV-2: associations with post-vaccination infection and risk factors in two UK longitudinal studies.*12*10.7554/eLife.80428

Niu, L., Xu, Z., & Taylor, J. A. (2016). RCP: a novel probe design bias correction method for Illumina Methylation BeadChip. *Bioinformatics (Oxford, England), 32*(17), 2659–2663. 10.1093/bioinformatics/btw285

Xu, Z., Niu, L., Li, L., & Taylor, J. A. (2016). ENmix: a novel background correction method for Illumina HumanMethylation450 BeadChip. *Nucleic Acids Research, 44*(3)10.1093/nar/gkv907

Zhang, Z., Zeng, C., & Zhang, W. (2022). Characterization of the Illumina EPIC Array for Optimal Applications in Epigenetic Research Targeting Diverse Human Populations. *Epigenetics Commun, 2*10.1186/s43682-022-00015-9
